# The influence of sea ice on the detection of bowhead whale calls

**DOI:** 10.1038/s41598-022-12186-5

**Published:** 2022-05-20

**Authors:** Joshua M. Jones, John A. Hildebrand, Bruce J. Thayre, Ellen Jameson, Robert J. Small, Sean M. Wiggins

**Affiliations:** 1 Scripps Institution of Oceanography, University of California San Diego, La Jolla, CA 92093-0205 USA; 2 University of San Diego, 5998 Alcala Park, San Diego, CA 92110 USA; 3 Alaska Department of Fish and Game, 1255 W. 8th Street, Juneau, AK 99811-5526 USA

**Keywords:** Marine mammals, Acoustics, Ocean sciences

## Abstract

Bowhead whales (*Balaena mysticetus*) face threats from diminishing sea ice and increasing anthropogenic activities in the Arctic. Passive acoustic monitoring is the most effective means for monitoring their distribution and population trends, based on the detection of their calls. Passive acoustic monitoring, however, is influenced by the sound propagation environment and ambient noise levels, which impact call detection probability. Modeling and simulations were used to estimate detection probability for bowhead whale frequency-modulated calls in the 80–180 Hz frequency band with and without sea ice cover and under various noise conditions. Sound transmission loss for bowhead calls is substantially greater during ice-covered conditions than during open-water conditions, making call detection ~ 3 times more likely in open-water. Estimates of daily acoustic detection probability were used to compensate acoustic detections for sound propagation and noise effects in two recording datasets in the northeast Chukchi Sea, on the outer shelf and continental slope, collected between 2012 and 2013. The compensated acoustic density suggests a decrease in whale presence with the retreat of sea ice at these recording sites. These results highlight the importance of accounting for effects of the environment on ambient noise and acoustic propagation when interpreting results of passive acoustic monitoring.

## Introduction

Passive acoustic monitoring (PAM) is an important tool for studying marine mammal seasonal distribution, migration, and behavior, especially in remote locations such as polar regions, where sea ice cover and light limitations prevent ship access and visual surveys for much of the year^1–4^. PAM allows marine mammal population density estimation to help with stock assessment and management^5–8^. Density estimation requires knowledge of the distance between the source (calling animal) and the receiver (PAM sensor)^9^, but environmental factors can substantially alter the effective listening area around a recording location. Factors in the environment that can influence the propagation of underwater sounds include water column and seabed physical properties, bathymetry, sea state and sea ice coverage. These influences on acoustic propagation are specific to a hydrophone location and affect the detectability of recorded signals by altering the signal-to-noise ratio. Accounting for these factors is important for analysis of PAM detection time series and for understanding their relationships with habitat or other ecological variables.

Acoustic propagation modeling and ocean noise levels have been used previously to account for environmental effects on marine mammal sound detection^10,11^. For example, the estimated probability of detecting humpback whale calls in the 0.2–1.8 kHz frequency range within a 20 km radius of three recording locations off California differed by up to 60% due to site-specific effects of bathymetry and seabed properties on sound transmission^10^. At one location, detection probability was also reduced by > 50% within the 20 km radius due to an increase in ocean noise levels by 10 dB^10,12^. When propagation modeling is combined with measurements of ambient noise and instrumental self-noise levels, it is possible to compensate for time-varying acoustic detection probability and thereby to improve estimates of acoustic density^12^.

In Arctic waters, rapid decreases in sea ice and increases in human activities, such as commercial shipping and mineral extraction, motivate the need to monitor marine mammal populations^13^. Bowhead whales (*Balaena mysticetus*) are closely associated with sea ice and have been the subject of decades of research efforts^4,14^, especially in the Bering-Chukchi-Beaufort (BCB) Seas where bowheads are harvested for subsistence by Inupiat communities^15^ and where exploration and development of offshore petroleum has raised concerns about underwater noise impacts^16^. Reductions in Arctic sea ice have the potential to significantly impact bowhead whales, both by changing their foraging environment and by exposing them to anthropogenic activities such as ship traffic and commercial fishing^17^.

**Figure 1:**
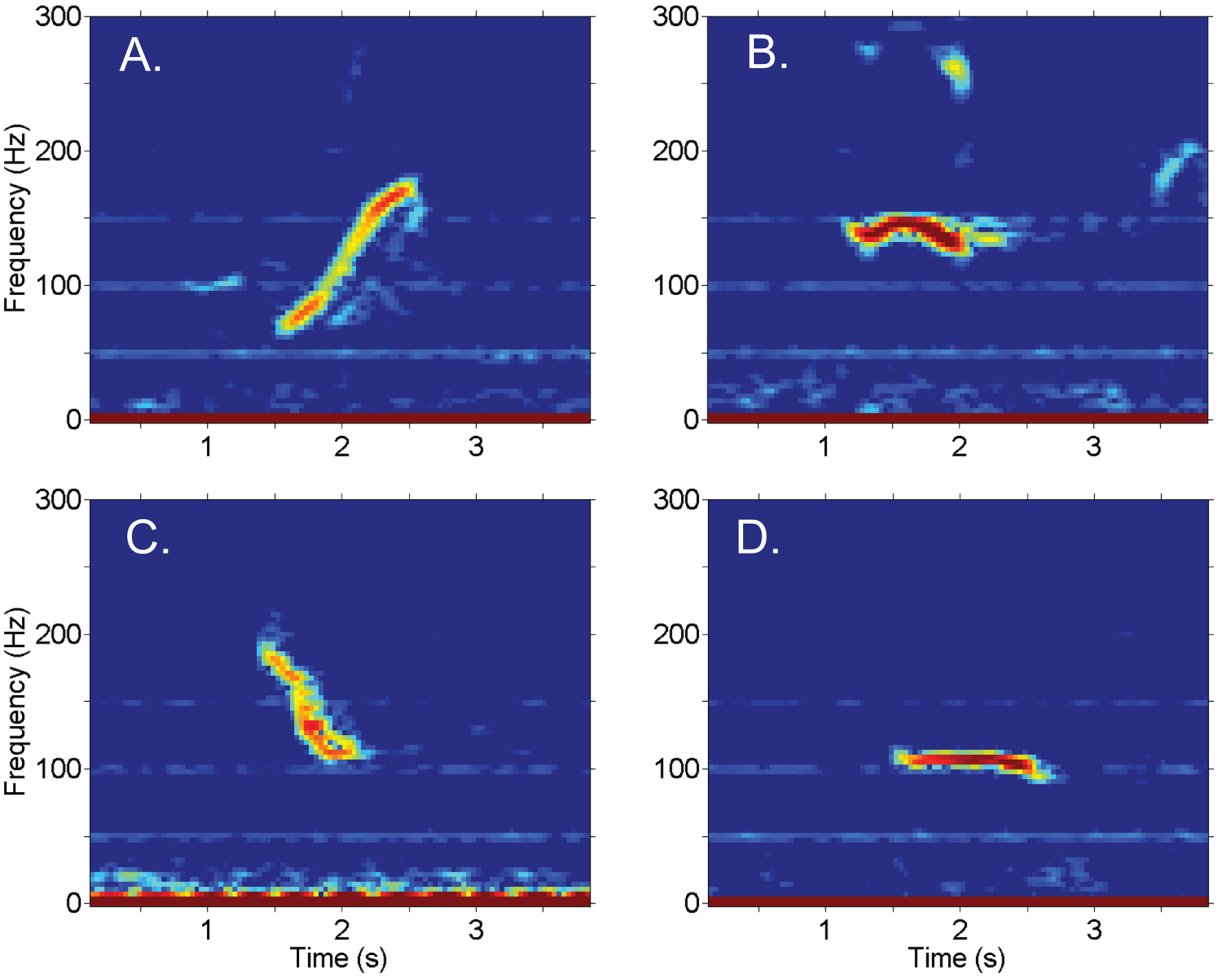
Spectrograms of bowhead FM calls including: (**A**) upsweep, (**B**) undulating n, (**C**) downsweep, and (**D**) constant. Undulating u call type not shown. Sample rate: 2000 Hz, FFT length: 550 samples, window overlap: 87%, window type: Hanning.

Bowhead underwater sound production includes a great variety of sounds, but often consists primarily of simple, narrow-band, frequency-modulated calls (FM calls), usually less than 200 Hz Fig. 1^14,18,19^. These relatively simple bowhead calls have been used to indicate their seasonal presence in PAM-based studies across much of the BCB bowhead range^4,18,20,21^, contributing to knowledge of their seasonal movements and distribution.

In the Arctic, the presence of sea ice influences acoustic propagation and ambient noise^22,23^. Sea ice strongly scatters sound in ways that are dependent upon ice thickness and underside roughness, and upon the frequency of the sound. Across a 100 Hz frequency band that includes most bowhead FM calls (80–180 Hz), there is substantially higher attenuation when sea ice cover is present than when compared to open-water conditions^24–26^. Previous experiments in the Arctic have found scattering from sea ice cover to contribute 10 to 15 dB of attenuation at frequencies between 70 and 200 Hz with sound paths at surface grazing angles of 10–20°^27–29^.

Interaction with the sea bottom is another factor influencing the propagation of sound, with bottom interactions increasing for a given frequency as water depth of the source or receiver decreases^30^. At a site on the Beaufort-Chukchi continental slope (receiver depth 328 m), airgun pulses from a deep-water survey (> 3000 m water depth) were recorded more than 500 km away, whereas from a shallow-water survey (< 100 m water depth) they were detected only up to ~ 100 km and experienced transmission losses much greater than the typical spherical spreading, due to interaction with the seafloor^31^.

Arctic ambient noise levels may vary over time due to sea ice, wind, currents and surface waves changing on seasonal and shorter time scales^32–34^. Mean ambient noise levels in the bowhead whale FM call frequency band (80–180 Hz) are highest during fall open-water conditions and lowest during spring ice-covered conditions^33^. Sound pressure spectrum levels increase with wind speed (u) by ~ 20 log_10_(u) in open-water^35^ but by only ~ 5 log_10_(u) with 75–100% ice-cover^33^. Episodic events in the sea ice layer (motion, deformation and fracturing) also create underwater noise^34,36^. Seismic surveys can increase noise levels significantly; for example, by 3–8 dB in the 80–180 Hz band during extended seismic survey periods^33^. Ocean currents can also cause flow-induced oscillations of the hydrophone (strum) in the 1–500 Hz band, resulting in instrumental noise, rather than true ocean ambient noise^37^.

**Figure 2:**
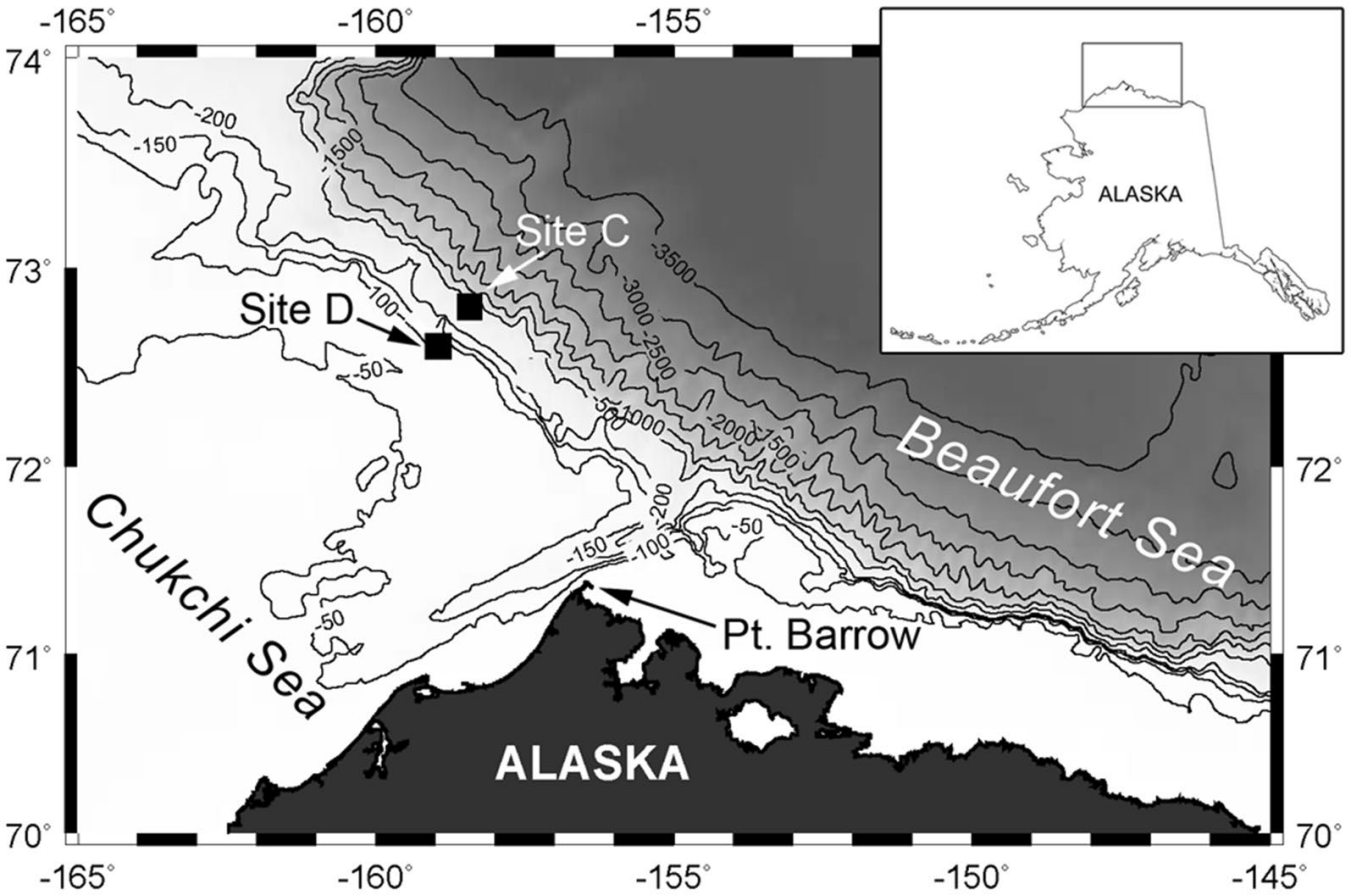
Acoustic recorder deployment sites near Utqiaġvik (Barrow, Alaska, USA) from 2012 through 2013. Contour depths in meters with darker shading indicating deeper depths. Sites C (slope) and D (shelf) at depths 320 and 100 m, respectively. Map created using M_Map mapping package (www.eoas.ubc.ca/~rich/map.html) for Matlab (MathWorks Inc., Natick, MA).

In this study, we use underwater acoustic modeling to develop a site-specific and time-dependent compensation applied to time series of bowhead whale acoustic detections at recording sites in the northeast Chukchi Sea outer shelf and continental slope (Fig. 2). The compensated bowhead whale acoustic detections provide a quantitative assessment of whale presence during changing environmental conditions, such as during the transition between open-water and sea ice-cover.

## Results

### Model for acoustic propagation and detection probability

**Figure 3:**
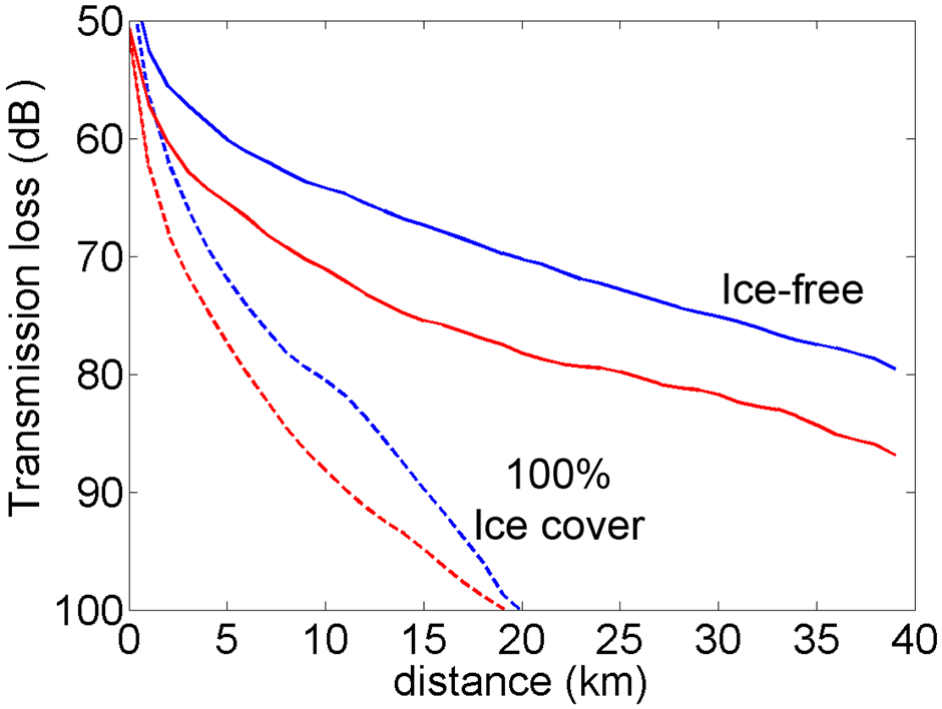
Average transmission loss for ranges from 0 to 40 km at the shelf (blue) and slope (red) sites in open water (solid lines) and ice-covered (dashed lines) conditions.

Sound transmission losses in the frequency band of bowhead whale FM calls are substantially higher in the presence of ice-cover than for open-water conditions (Fig. 3 and Table 1). This suggests that there will be long-range propagation of 80–180 Hz signals during open-water but rapid attenuation during ice-cover. At a range of 20 km, losses are 25–30 dB greater during ice-cover, consistent with the expectation that interaction with an ice-covered surface results in scattering of acoustic energy.

**Table 1:** Average modeled sound transmission loss (TL) for 80–180 Hz sounds traveling in all directions (All azimuths), toward shallower water (Upslope), and toward deeper water (Downslope) at the slope and shelf recording sites. Values are average and standard deviation of all 1 km^2^ model locations within radii < 20 km, 20–40 km, and for all locations < 40 km. Number of locations included in statistical computation given as n(locs).

Direction of propagation (deg T)	Radius	n (locs)	Slope site TL_avg_ (dB)		Shelf site TL_avg_ (dB)	
			Ice-covered	Open-water	Ice-covered	Open-water
All azimuths	< 20 km	1256	91.1 ± 8.3	73 ± 5.8	85.9 ± 11.1	65.7 ± 3.9
0–360°	20–40 km	3654	108.7 ± 7.5	82.3 ± 5.1	117.6 ± 21.2	75.3 ± 3.4
	< 40 km	4910	104.2 ± 10.9	79.9 ± 6.7	109.5 ± 23.6	72.8 ± 5.5
Upslope	< 20 km	158	92.1 ± 8.2	69.8 ± 3.2	90.8 ± 13.6	64.6 ± 3.1
22.5–67.5°	20–40 km	454	116.2 ± 9.1	78.2 ± 2.8	143.3 ± 14.5	73.3 ± 2.4
	< 40 km	612	110 ± 13.8	76.1 ± 4.7	129.8 ± 27.1	71 ± 4.6
Downslope	< 20 km	158	92.5 ± 8.9	78.4 ± 6.5	82.7 ± 6.6	67.7 ± 4
202.5–247.5°	20–40 km	454	105.9 ± 4.1	88.5 ± 2.5	103.2 ± 7	78.5 ± 3
	< 40 km	612	102.4 ± 8.2	85.9 ± 5.9	97.9 ± 11.4	75.7 ± 5.8

**Figure 4:**
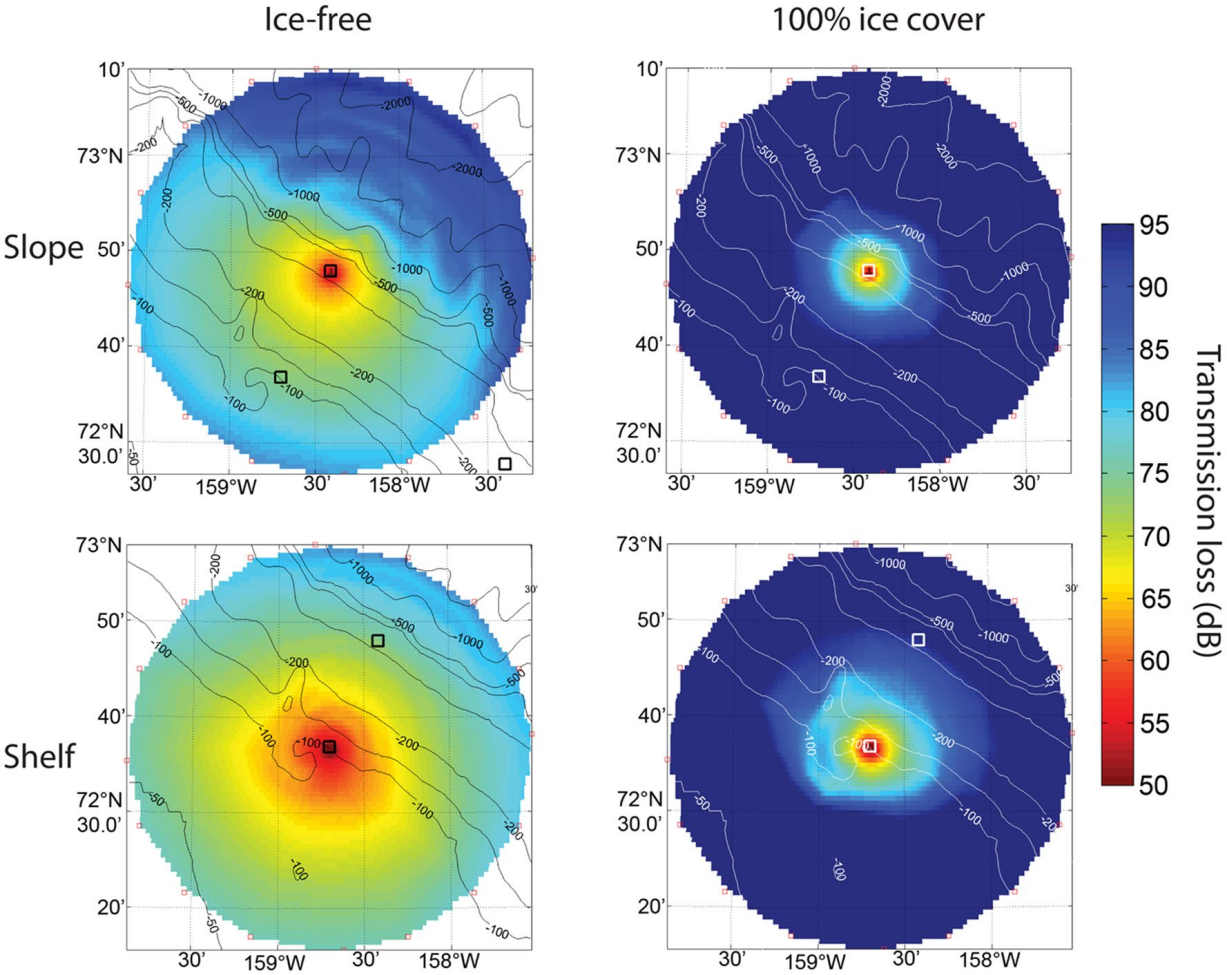
Transmission loss in dB for: open-water (left panels) and ice-covered (right panels) conditions for Chukchi slope (upper panels) and shelf (lower panels) sites with receivers at 320 and 88 m, respectively. Contour lines in m.

Averaged across all azimuths at distances from 0 to 20 km, propagation losses are greater at the slope site than at the shelf site in both ice states (Table 1), partly due to the slope site’s greater exposure to deep water (Fig. 4). The slope site has higher losses than the shelf site at 0–20 km distance in both the upslope and downslope directions with ice cover and in the downslope direction in open water. Two exceptions occurred in the overall pattern of propagation losses with changing depth at ranges of 0–20 km. There was little difference in upslope propagation between the two locations in open water. With ice cover, average 0 to 20 km upslope and downslope losses were also not substantially different at the slope site.

At longer distances of 20–40 km from both sites during ice cover, sounds traveling upslope toward shallow water have higher predicted propagation loss than sounds traveling downslope toward deeper water (Table 1). This spatial pattern of propagation losses at longer distances is reversed during open water. Down slope propagation losses from 20 to 40 km distance are higher relative to sounds traveling upslope during open-water conditions. Upslope propagation results in more surface interactions with increasing distance, hence, more loss in the presence of ice-cover, whereas downslope propagation results in less surface interaction, hence avoiding the impact of ice-cover scattering.

**Figure 5:**
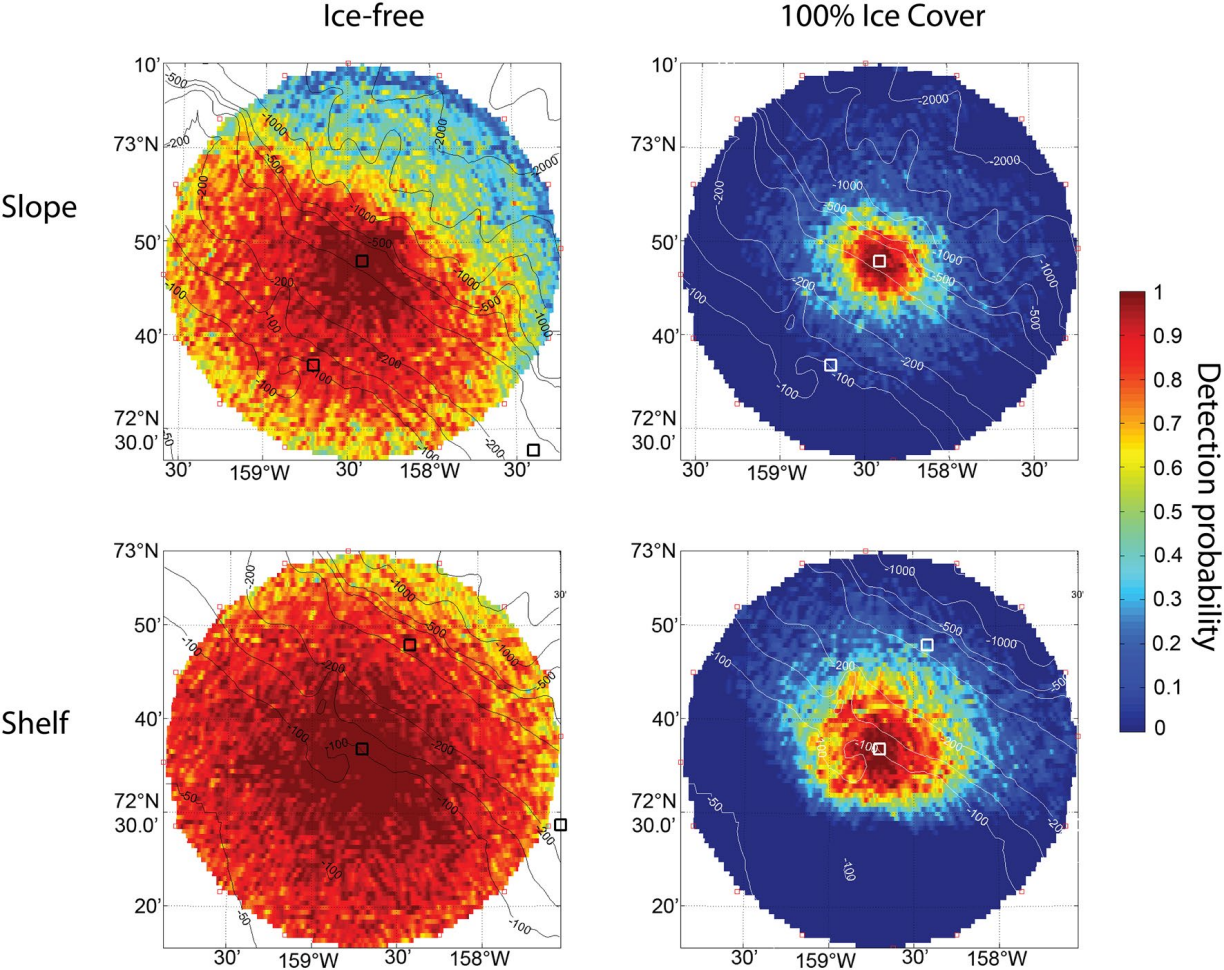
Detection probability for open-water (left panels) and ice-covered (right panels) conditions for slope and shelf recording sites (upper and lower panels) with 70 dB re 1 μPa^2^ noise spectrum level in the 80–180 Hz band.

Average detection probabilities for calls follow similar patterns to sound transmission loss (Fig. 5 and Table 2). Detection probability is substantially reduced in the presence of sea ice when compared to open-water; little or no detection is possible beyond 20 km in ice-cover, whereas calls are readily detected even at 40 km in open-water. Detection probability across all azimuths within a 40 km radius of each site is improved on the shelf compared to the slope in ice-free and ice-covered conditions.

**Table 2:** Average simulated detection probability (DP) with 70 dB re 1 μPa^2^ noise spectrum level in the 80–180 Hz band for sounds traveling in all directions (All azimuths), toward shallower water (Upslope), and toward deeper water (Downslope) at the slope and shelf recording sites. Values are average and standard deviation of all 1 km^2^ model locations within radii < 20 km, 20–40 km, and for all locations < 40 km. Number of locations included in statistical computation given as n(locs).

Direction of propagation (deg T)	Radius	n (locs)	SlopesiteDP_avg_ (%)		ShelfsiteDP_avg_ (%)	
			Ice-covered	Open-water	Ice-covered	Open-water
Allazimuths	< 20 km	1256	30.5 ± 25.7	85.9 ± 14.1	49.3 ± 30.1	97.2 ± 3.7
0–360°	20-40 km	3654	1.9 ± 3.7	61.5 ± 20.3	4.9 ± 8.5	83.8 ± 10.6
	< 40	4910	9.4 ± 18.5	68.1 ± 21.7	16.5 ± 25.9	87.3 ± 11
Upslope	< 20 km	158	27.9 ± 24.3	93.7 ± 5.3	38.6 ± 34	98 ± 2.6
22.5–67.5°	20-40 km	454	0.4 ± 1.6	77.2 ± 10.8	<0.1 ± <0.1	88.2 ± 6.9
	< 40	612	7.8 ± 17.5	81.6 ± 12.2	10.3 ± 24.5	90.8 ± 7.4
Downslope	< 20 km	158	26.6 ± 27	71.3 ± 19.1	57.3 ± 21.1	95.1 ± 4.9
202.5–247.5°	20-40 km	454	2.4 ± 3.9	38.6 ± 12	6.9 ± 10.3	75.2 ± 11.1
	< 40	612	8.7 ± 17.7	47.1 ± 20.2	20.1 ± 26.2	80.4 ± 13.2

**Figure 6:**
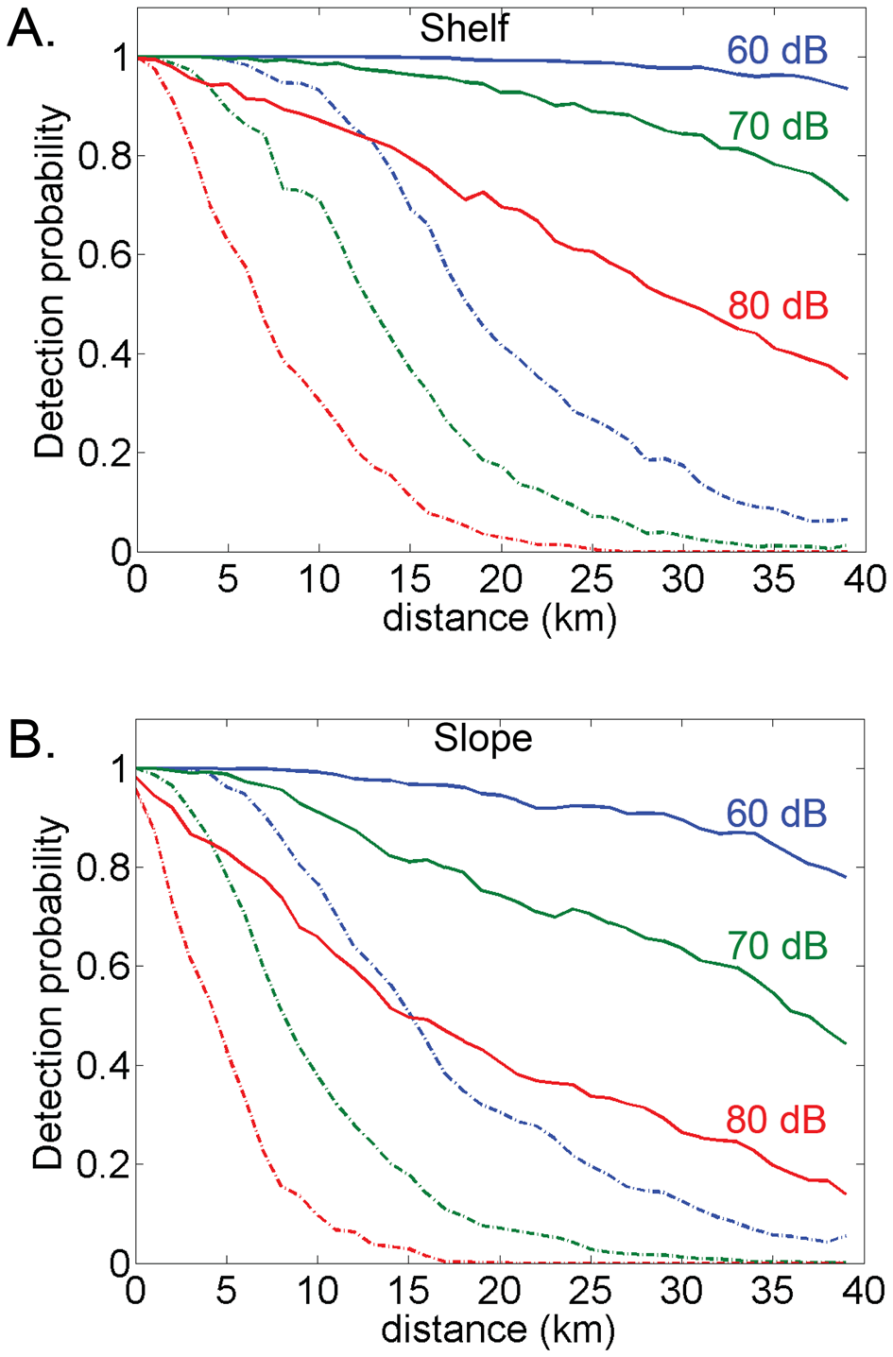
Detection probability as a function of distance for (**A**) shelf and (**B**) slope sites at ambient sound pressure level 60, 70, and 80 dB re 1 μPa^2^ (blue, green, and red lines) with open-water (solid line) and ice-covered (dashed line) conditions.

Averaging detection probability across azimuth also gives detection probability as a function of distance for each noise level and ice state (Fig. 6). The probability of call detection falls off with range from the recording site, more steeply with increasing ambient noise level, both with and without sea ice (Fig. 6). Detection probability decreases slowly with range in open-water but decreases rapidly for ice-covered waters.

**Figure 7:**
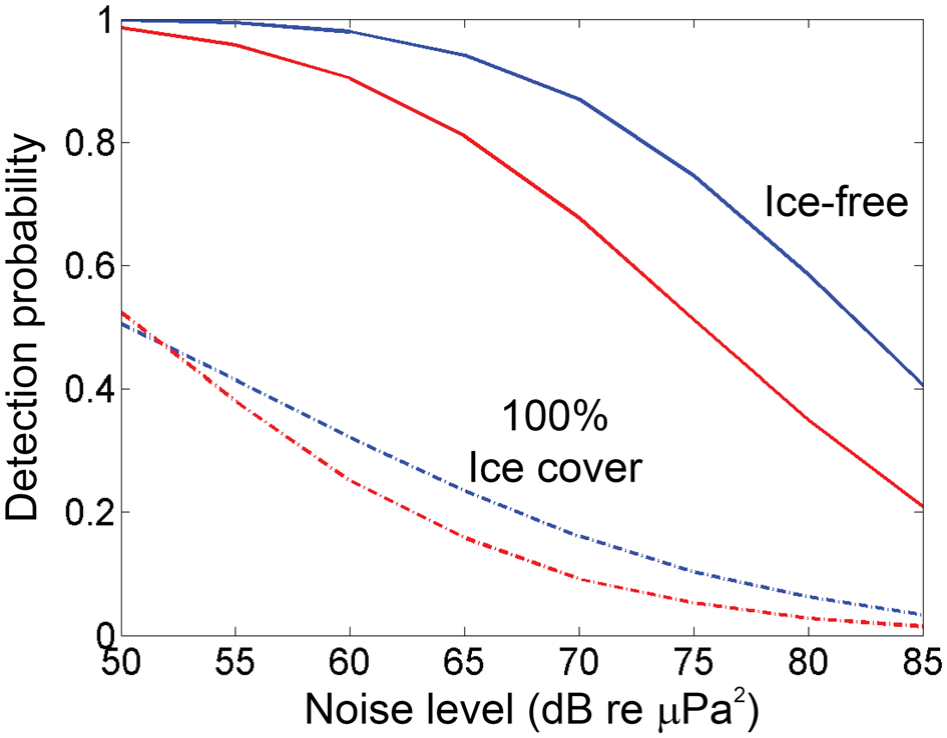
Modeled average detection probability (P̂) within a 40 km radius as a function of noise level in the 80–180 Hz band for each ice state and site. Blue and red lines represent the shelf and slope sites, respectively in open-water (solid line) and ice-covered (dashed line) conditions.

Mean detection probability is modeled as a function of noise level for each site and ice state within the 40 km radius in Fig. 7. With sea ice-cover, average detection within 40 km is substantially lower than with open-water at both sites and all noise levels; the average detection probability is 20–60% lower with the sea ice layer. Detection is somewhat higher on the shelf than the slope, but with the sea ice layer (dashed lines in Fig. 7), average detection probability is similar at both locations and ranged from ~ 50% at the lowest noise levels (50 dB re 1 µPa^2^) to less than 5% at the highest noise levels. With ice cover, the effect of additional noise on detection probability is greatest at distances 5–20 km, while the effect of added noise is greatest at > 20 km distance in open water. For example, at distance 10 km on the slope site, increasing noise from 60 to 80 dB reduces detection probability from 80 to ~ 5%. In open water, this noise increase reduces detection probability at a 10 km radius from > 95 to ~ 60%. Overall, adding noise results in substantially lower detection probabilities and a decreased detection area at shorter radial distances with ice cover and longer radial distances in open water.

In both open-water and ice-covered states, the spatial patterns in detection probability also correspond to bathymetry (Fig. 5). At shorter distances from 0 to 20 km and typical noise conditions of 70 dB re 1 µPa^2^ spectrum level in the 80–180 Hz band, detection probabilities in open water are > 90% for calls originating upslope from both sites and for sounds originating downslope from the shelf site (Table 2). Under the same open-water and noise conditions, 0 to 20 km average detection probability is reduced to approximately 70% for sounds originating downslope from the slope site (Table 2). With ice cover, 0–20 km average detection probabilities are similar in the downslope and upslope directions at the slope site, but higher in the downslope than upslope direction at the shelf site (Table 2). The greater probability of detecting bowhead calls at 0–20 km distances downslope from the shelf with ice cover corresponds to the approximately 10 dB lower transmission losses predicted for sounds traveling into deeper water from the shelf site than the slope site with ice cover (Table 1).

At longer distances of 20–40 km in typical noise conditions, average upslope detection probabilities are > 77% at both sites in open water, whereas the longer-range downslope detection probability is substantially lower at the deeper slope site (Table 2). This pattern disappears or is reversed with ice-cover. While detection probabilities are low in all directions at 20–40 km, there may be somewhat greater probability of detection downslope than upslope from the recorder, particularly at the shelf site.

### Observations of bowhead acoustic occurrence

**Figure 8:**
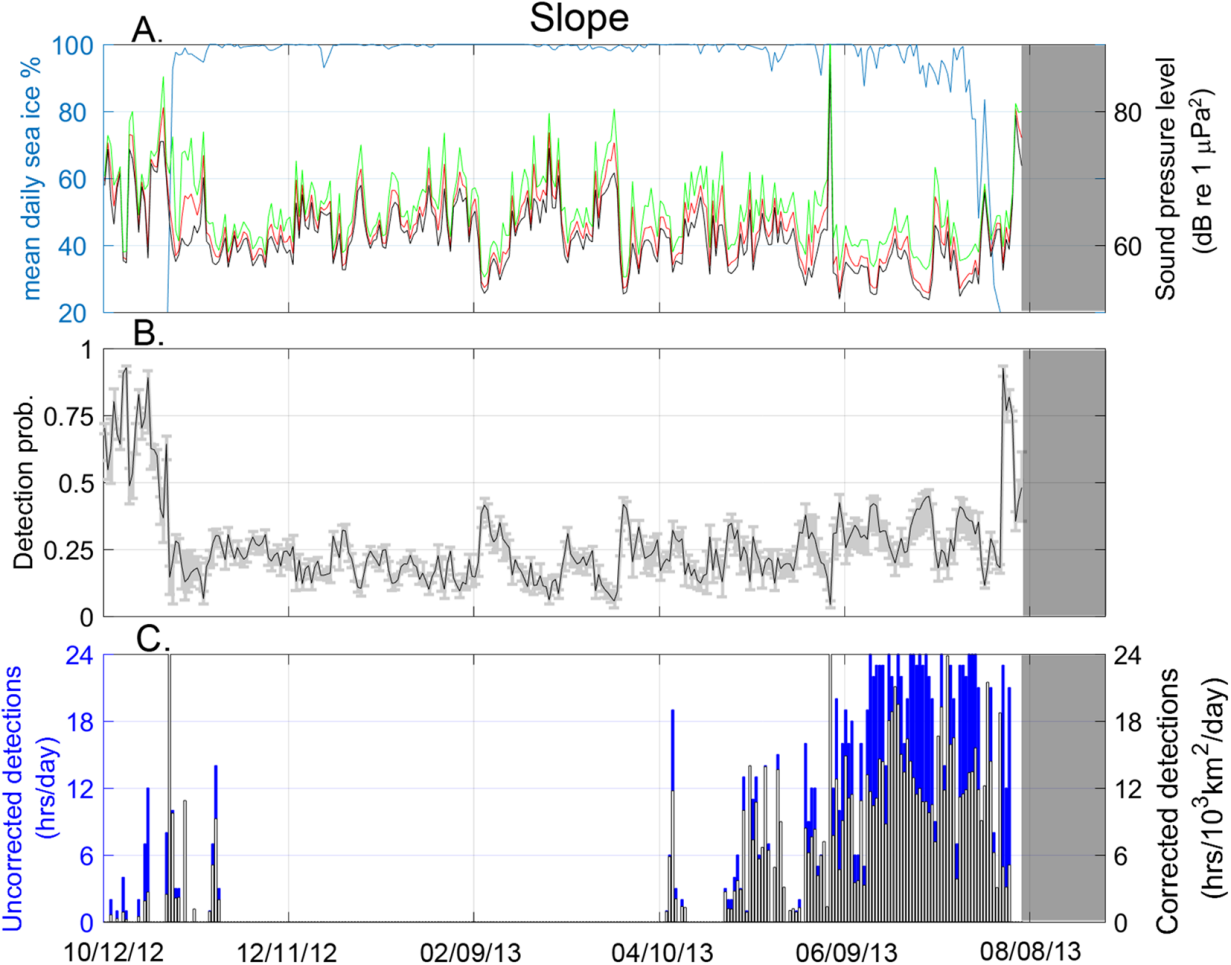
Slope site (**A**) Average daily ambient noise levels in daily 10th 50th, and 90th percentiles (black, red, and green lines, respectively) for 80–180 Hz frequency band and mean daily sea ice concentration (blue line from October 2012 to October 2013. (**B**) Daily detection probability within a 40 km radius, gray bars show range for the 90th and 10th percentile noise level. (**C**) Bowhead whale call detections as uncompensated daily detection hours (blue bars) and compensated detection density (black and white bars). Gray shaded areas indicate period of no recording.

**Figure 9:**
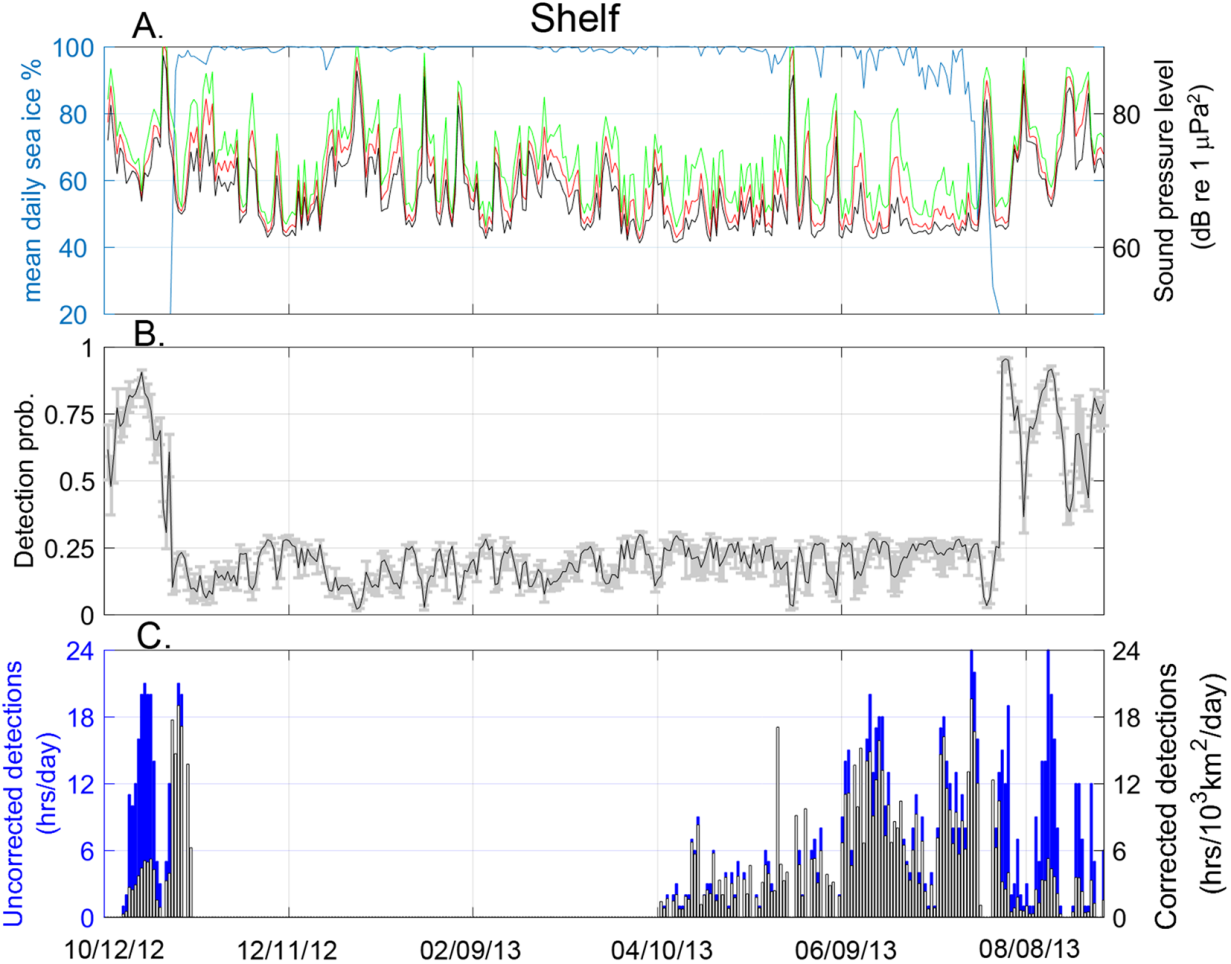
Shelf site (**A**) Average daily ambient noise levels in daily 10th 50th, and 90th percentiles (black, red, and green lines, respectively) for 80–180 Hz frequency band and mean daily sea ice concentration (blue line from October 2012 to October 2013. (**B**) Daily detection probability within a 40 km radius, gray bars show range for the 90th and 10th percentile noise level. (**C**) Bowhead whale call detections as uncompensated daily detection hours (blue bars) and compensated detection density (black and white bars).

In October 2012, open-water was present at both the slope (Fig. 8) and shelf (Fig. 9) recording sites but by early November 2012 both sites experienced a rapid transition to nearly complete ice-cover. The ice remained at these sites for ~ 8 months, until July 2013, at which time there was an approximately one-week transition to open-water conditions.

Ambient noise levels in the 80–180 Hz frequency band were higher by ~ 10 dB during periods with little or no ice coverage at both the slope (Fig. 8) and shelf (Fig. 9) sites. In addition, average noise levels during open-water at the slope (63 ± 6 dB re 1 μPa^2^) were ~ 7 dB lower than those at the shelf (70 ± 7 dB re 1 μPa^2^) site due to current-induced strumming of mooring hardware at the shelf recorder.

The detection probability for bowhead FM calls was significantly higher during open-water than during ice-cover at both sites (Figs. 8 and 9). At the slope site during open-water the detection probability was 68 ± 10% and fell to 10 ± 5% during ice-cover. Likewise, at the shelf site open-water detection was 85 ± 10% and ice-cover was 18 ± 5%. This suggests that bowhead calls are ~ 3 times more likely to be detected during open-water periods, owing to the signal attenuation with ice-interactions.

Bowhead whale FM calls were detected at both sites in the fall, during the time of ice formation, and in the spring, well before and following ice breakup (Figs. 8 and 9). Calls were present during most open-water periods, during spring and early summer high ice cover, and during freeze-up in October and November. From April through July, as sea ice concentration varied between 90 and 100%, bowhead detections increased at both sites, with some variability on daily and weekly time scales. The detection rates appear comparable before and after transitions between open-water and ice-covered conditions at both sites, for instance, at the shelf site high rates of detection (> 18 h/day) were experience both in June (ice-covered) and August (open-water) conditions.

Compensating the detection time series using Eq. 5 provides acoustic occurrence relative to detection area (detection hrs/1000 km/day). The compensated density of acoustic occurrence reduces the estimate for bowhead presence during open-water periods, for instance, in the period before freeze-up at the shelf site in October 2012 (Fig. 8). This occurs primarily due to the effect of lower sound transmission losses in open water, which raises detection probability and increases the effective listening area around the recording sites. Even at relatively high noise levels (e.g. 80 dB), the detection probabilities are greater in open water at distances 20–40 km than during the lowest noise levels (e.g. 60 dB) with ice cover (Fig. 6). At both sites, there was a decline in estimated acoustic density following the breakup of sea ice (Fig. 8 and Fig. 9) that was not reflected in the uncompensated detection time series.

## Discussion

In the Arctic, seasonal sea ice cover dramatically decreases the range over which bowhead whale FM calls can be detected. With the presence of ice-cover, sound attenuates to undetectable levels over much shorter distances than in open-water. This has a large effect on call detection probability*,* yielding a bimodal distribution of detection probability over the course of a year, whereby calls are ~ 3 times more likely to be detected during open-water conditions. Without compensating for the effects of ice-cover on sound propagation, acoustic presence is overestimated during open-water periods and underestimated when sea ice is present. This is despite the increase in ambient noise levels during open-water. The impact of sound attenuation by ice-cover on call detection is greater than the decrease in signal-to-noise owing to higher open-water noise levels. The compensation method presented here, therefore has the effect of normalizing the estimates of detection density across periods of varying ice-conditions, as well as between the two sites, including differences in environmental and instrumental characteristics. This presents the possibility of comparing detection densities between sites to provide quantitative estimates of bowhead presence.

The presence of a sea ice layer dramatically increases transmission loss (TL), consistent with previous studies of Arctic long-range sound propagation^29^. Diachok^25^ found TL of 95 dB at 200 Hz from 40 km with sea ice, compared with a theoretical prediction of 76 dB with open-water conditions. For the slope site, TL was 105 dB at 40 km with ice cover and 76 dB in open-water. The shelf site had higher TL of 110 dB at 40 km range for ice-cover and TL of 68 dB in open-water. The increased TL for the shelf site with sea ice-cover is likely due its shallower depth, resulting in more reflections off the seafloor which then interact with the underside of the sea ice at the surface. The low up-slope and along-slope TL at the shelf site may also be related to less interaction with the sea bottom and the surface. Due to the sound speed profile and deeper water, the slope site is located in an acoustic waveguide during open-water^31^.

In the arctic, increasing sound speed with depth results in rays that are upward refracted and interact with the surface, making surface scattering an important parameter for sound propagation. Surface scattering strength increases as a function of wind speed during open-water conditions^38^. Our model uses an RMS roughness of 1.38 m for the sea surface in open-water, which would correspond to typical wind speeds (~ 5 m/s) observed in the study area^33^. Higher wind speeds do occur during open-water (> 15 m/s) and would result in greater surface roughness and increased ambient noise levels. At higher wind speeds, scattering strength increases for bowhead whale call frequencies at grazing angles up to 25°^39^. The effect of surface roughness might reduce the high detection probability we predict for open-water conditions and should be incorporated into future models. Scattering of acoustic energy is also affected by the number and depth of ice keels under pressure ridges^25^ and in the marginal ice zone^40^. Future models should develop additional parameters to account for these factors in acoustic propagation.

Applying the detection density function (Eq. 5) to uncompensated detections had the effect of normalizing acoustic occurrence across the two sites that differ in both sound propagation environment and noise characteristics. The uncompensated daily call occurrence suggests a greater presence of bowheads at the slope than the shelf site during ice-cover, with many June and July days having nearly continuous (24 h) presence at the slope site but with few days above 12 h of presence at the shelf site. Examining the acoustic density at both sites (Figs. 8 and 9) this apparent difference in bowhead presence between the two sites disappears. With the model-derived compensation applied, the density of acoustic occurrence at the two sites is comparable during the same time period, with both sites having a density index of ~ 7.5 h/10^3^km^2^/day within a 40 km radius.

Examining bowhead acoustic density with respect to sea ice, the annual patterns of presence are similar at both sites, with whales first appearing in April during full ice-cover and again approximately two weeks before the onset of ice formation through freeze-up. A sustained period of acoustic density occurs at the shelf site prior to freeze up in the fall and increases during ice formation. This fall density of occurrence does not appear as pronounced at the slope. Both sites exhibit peaks in acoustic density in June and July, which decreases around the onset of sea ice break-up. This pattern does not appear in the uncompensated acoustic detection time series and is consistent with satellite telemetry results showing seasonal movements of bowheads extending to areas of their range outside of previous survey coverage for their abundance and distribution^41^.

How might bowhead whales have optimized the propagation of their FM calls based on the environment of the Arctic? The frequency for optimum (minimum loss) acoustic propagation in shallow open-water is dependent on depth. In temperate oceanographic conditions, optimum propagation frequencies are about 220–50 Hz for depths from 100 to 300 m, respectively^30^. Optimum frequencies decrease in the Arctic due to the upward refracting sound speed profile, increasing sound transmission loss through surface interactions^31^. Loss to the seafloor becomes greater at lower frequencies^30^ and is expected to be significant in the thick sediment layers of the Chukchi Sea. This may increase the lower end of the optimum frequency band relative to other areas. The combined effects of these factors may act to narrow the bandwidth of frequencies that propagate best within the study area, presumably corresponding to the 80–180 Hz band used for bowhead FM calls.

Characteristics of bowhead whale calls may change over time or with environmental conditions and ambient sound levels; and this potential variability may need to be incorporated into future development of acoustic density estimation for bowhead whales. Minimum and peak frequencies of bowhead calls in the Beaufort Sea decreased during 2008–2014, with an increasing proportion of calls below 75 Hz^42^. Thus, frequencies below 80 Hz should be evaluated for inclusion in sound propagation modeling for bowhead acoustic density. While this study does not distinguish between call categories beyond a focus on non-song FM calls, some call categories such as “complex” calls may be shifting to lower frequencies to a larger degree than others^42^. Where call categories have different frequency characteristics, the proportion of calls by category may also be important for improving future detection probability and density estimation.

Detection of acoustic signals from bowhead whales in Arctic shelf and slope waters are subject to environmental factors that affect the transmission of the sounds and factors that affect the levels of ambient noise. To help interpret results from passive acoustic monitoring detections, it is necessary to address these factors and account for them. Applying this compensation method to multi-year time series of acoustic data will substantially improve the use of passive acoustic monitoring in estimating abundance and distribution of marine species and to investigate ecological relationships with environmental factors, such as sea ice.

## Methods

### Acoustic recordings

High-frequency Acoustic Recording Packages (HARPs) were deployed at two locations along the northeast Chukchi Sea shelf (site D) and slope (site C) between October 2012 and October 2013 (Fig. 2). These instruments recorded at a sampling rate of 200 kHz (effective bandwidth 10–100 kHz) with a schedule of 10 min recording every 15 min. The hydrophone consisted of a low-frequency stage with six cylindrical transducers (Benthos AQ-1; http://teledynebenthos.com) with a combined sensitivity of − 193.5 dB re: V/µPa and preamplifier gain of 50 dB, and a high-frequency stage with a single spherical transducer (ITC 1042; http://www.piezo-kinetics.com) with a relatively flat (± 2 dB) sensitivity of − 200 dB re: V/µPa from 1 to 100 kHz^43^. All acoustic recordings were converted into an adapted wav file format (XWAV) for analysis. To minimize computational requirements, XWAV files were decimated by a factor of 20 using an eighth-order Chebyschev type I filter (10–5000 Hz). Analyses were conducted using the *Triton* program, based on MATLAB (MathWorks Inc., Natick, MA), to calculate and display long-term spectral averages (LTSA) and spectrograms, to perform audio playbacks, and to log call detections^43^.

### Call detections

Data were visually scanned as 30-min decimated LTSAs (FFT length: 500, no overlap, frequency range 10–500 Hz) for bowhead whale FM calls, using previously described acoustic behavior^14,18^ to compare with calls detected in the recordings. To simplify identification, only the simple, frequency-modulated non-song type calls were accepted as initial detections of bowhead whales

Figure 1 Following detection in the 30-min LTSA window, a spectrogram (FFT length: 1000, overlap 60%, frequency range 5–500 Hz) of 60 s or less was inspected to check the identity of the call. A minimum of one call was logged for each hour in which calls were taken to be present, providing hourly resolution. Finally, spectrograms of the logged calls were re-inspected for errors, and misidentified calls were removed.

### Detection probability

To convert acoustic occurrence to a model for density, the area monitored must be estimated; for a single sensor this is done by estimating the detection probability as a function of horizontal range from the hydrophone^44^. Detection probability for calling whales can be estimated using a Monte Carlo method^11,45^ with adaptation for site-specific sound propagation^12^. Detection probability ($\hat{P}$) for an area within the maximum detection radius ($w$) can be calculated as a function of range ($r$) and azimuth ($\theta$) from the recording location as follows,

1 $$\hat{P} = \mathop \smallint \limits_{0}^{w} \mathop \smallint \limits_{0}^{2\pi } \rho \left( {r,\theta } \right)g\left( {r,\theta } \right)rdrd\theta$$

where $\rho \left( {r,\theta } \right)$ is the probability density function for whale locations and $g\left( {r,\theta } \right)$ is the detection probability. If an homogeneous distribution of whales within the detection area is assumed, then the probability density function becomes $\rho \left( {r,\theta } \right)$ = 1/π*w*^2^. Detection probability is then estimated using a parametric model for $g\left( {r,\theta } \right)$ that includes characteristics of bowhead whale acoustic behavior including source level and depth for calling whales, and the seasonal acoustic environment, including time-varying ambient noise levels, recording site properties, and sound propagation modeling.

Source levels for bowhead whale FM calls (Fig. 1) have been reported with a roughly normal distribution at 157 ± 10 dB re 1 μPa @ 1 m^46^ or similarly 161 ± 9 dB_RMS_ re: 1 μPa @ 1 m^47^. Some of the variability in bowhead call source level may be related to their directivity pattern for radiated energy^48^, with a source level for whales traveling toward the receiver ~ 4–5 dB higher than for whales moving away. For the purpose of modeling detection probability, random orientation of the animals with respect to the receiver was assumed, and a constant calling depth of 26 m was chosen, as estimated by Thode et al.^47^ from localizing calls in the Beaufort Sea.

To choose a signal-to-noise threshold appropriate for detection probability simulation and modeling, an additional analysis was performed for a subset of calls from each site using every detectable call for: (1) the last one-week period before sea ice breakup with mean weekly ice concentration greater than 90% (ice-covered), (2) the week centered on the last day with 50% sea ice concentration before open-water (transitional), and (3) the last full week with sea ice no greater than 10% (open-water). The peak-to-peak (p-p) and root mean square (RMS) received sound pressure level for each call were calculated as follows:

2 $$RL_{p - p} = 20\log_{10} \left( {\max (P\left( t \right) - \min (P\left( t \right)} \right)$$

3 $$RL_{rms} = 20\log_{10} \sqrt {\frac{1}{T}\mathop \smallint \limits_{0}^{T} P^{2} \left( t \right)dt}$$

where pressure, *P*, is evaluated over the duration, *T*, in which the energy is between 5 and 95% of the total integrated energy in the band 80–180 Hz. To estimate ambient noise level, *p-p* received levels were calculated during the one second prior to each call. A simplified equation was used to determine the signal-to-noise ratio (SNR),

4 $$SNR \, = \, RL - NL$$

where RL is the *p-p* received level during the call and NL is the *p-p* level one second prior to the call. The detection threshold was estimated from the SNR distributions. A single threshold of 2 dB was chosen because 95% of the actual detected calls had a measured SNR above 2 dB. We assume that calls with RL less than 2 dB above the background level were not reliably detectable.

#### Recording site properties

The outer shelf and continental slope of the northeast (NE) Chukchi Sea is a transition zone between the shallow Chukchi Sea shelf (avg. depth ~ 50 m) and abyssal Canada Basin (avg. depth ~ 3600 m). The region is covered by seasonal sea ice, typically present from October through August, with substantial interannual variability in timing of ice formation and breakup^49^. Sea ice is primarily first-year with interannually variable proportions of thicker multi-year ice^50^. The water column characteristics in the NE Chukchi Sea slope vary seasonally and are strongly influenced by sea ice, extreme low air temperatures, sea ice melt, and Atlantic water present at depth^23^. A two-layer profile of temperature and salinity is often present, with a cold (~ − 2° C) relatively fresh upper layer during periods with ice cover, saltier relatively warmer underlying water column, and a weak thermocline^51,52^. The seafloor consists of relatively thick fine silt, sand, and clay, especially on the shallow shelf^53^.

**Figure 10:**
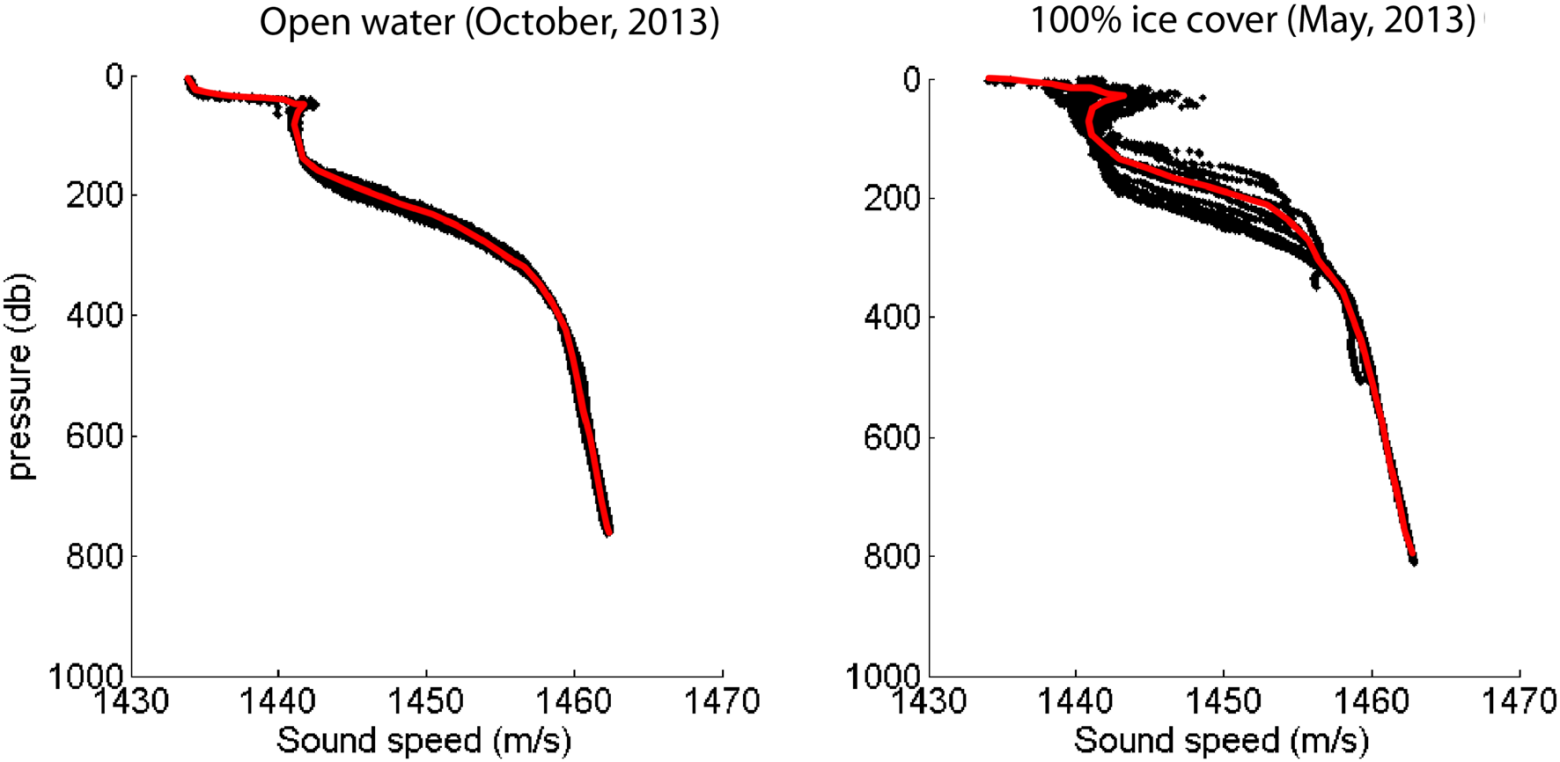
Sound speed profiles during 2013 October (left) and May (right). Black dots are measurements. Curve fit to the data (red line) is used as a representative sound speed profile. Open-water and ice-covered measurements were from the USCGC Healy and an Ice Tethered Profiler (ITP Misfigsion 62; Toole et al. 2011), respectively.

Data on the surface, water column, seafloor, and seabed were compiled (Table 3) to create a seasonal model for sound propagation at the recording sites. The hydrographic data were collected during complete ice cover by an ITP-62 Ice Tethered Profiler^54^ in May, 2013, and during open-water from the USCGC *Healy* in October, 2013. Sound speed profiles (Fig. 10) were calculated from pressure, salinity, and temperature using the Equation of State ^55^. A key feature of these profiles is that for both open-water and ice-covered conditions, a minimum in sound speed is observed at the sea surface, owing to the presence of cold and fresh water, resulting in upward-refracting ray paths and a surface sound transmission duct, with ample interaction of sound with the sea surface^56^.

**Table 3:** Acoustic model parameters.

Location	Parameter	Symbol	Value(s)	Units	Source
Sea ice	Sea ice thickness	*z*_*i*_	2.7	m	Goff, 1995
	Ice-water RMS roughness		1.38	m	(Ibid.)
	Ice-air RMS roughness		0.45	m	Gavrilov & Mikhalevsky, 2006
	Sea ice density	*ρ*_*i*_	0.89	g/cm^3^	Alexander et al. 2013
	Sea ice compressional speed	*c*_*i*_	3000	m/s	Gavrilov & Mikhalevsky, 2006
	Sea ice shear speed		1800	m/s	(Ibid.)
	Compressional wave attenuation		0.45	dB/l	(Ibid.)
	Shear wave attenuation		0.9	dB/l	(Ibid.)
Water column	Surface sound speed	*c*_*w*_	1435		ITP-62; HLY1303
	Seafloor sound speed	*c*_*w*_	1465		ITP-62; HLY1303
	Range-independent water depth	*z*_*w*_	2500	m	
	Density and sound speed profile shelf	*–*	–		ITP-62; HLY1303
	Density and sound speed profile basin	*–*	–		ITP-62; HLY1303
	Absorption		Negligible		Fisher & Simmons, 1977
Seafloor	Seafloor mean grain size	*d*	3.0, 3.6	µm	Xiangmei et al. 2015
	Depth (bathymetry)	*z*_*b*_	–		IBCAO v.3 1-min grid
Subbottom	Number of subbottom steps		5		Warner et al. 2015
	Step depths		2, 5, 10, 20, 40	m	(Ibid.)
	Layer compressional speed	*c*_*b*_	1465, 1555, 1605, 1750, 2200	m/s	(Ibid.)
	Layer shear speed				(Ibid.)
	Layer density	*ρ*_*b*_	1.49, 1.77, 1.87, 2.06, 2.2	g/cm^3^	(Ibid.)
Basement	Compressional speed	*c*_*u*_	2300	m/s	(Ibid.)
	Shear speed				(Ibid.)
	Density	*ρ*_*u*_	2.3	g/cm^3^	(Ibid.)

Bathymetric data were extracted from the International Bathymetric Chart of the Arctic Ocean ^57^ at a spatial resolution of 1 km and we adopted seafloor acoustic properties and a sub-bottom sound speed profile from previous studies on the Chukchi Shelf^53,58^.

Daily Advanced Microwave Scanning Radiometer 2 (AMSR2) sea ice maps^59^ were obtained from the University of Bremen (http://www.iup.uni-bremen.de:8084/amsr2data/asi_daygrid_swath/n6250/) and processed using Windows Image Manager (WIM) and Windows Automation Module (WAM) software^60^ to produce time series of mean daily Sea Ice Concentration (SIC) within 40 km of each recording site. WAM software was used to compute the daily arithmetic mean, variance, and median of the sea ice concentration as a percent of the total mask area. The 40 km radius was selected as a conservative detection range for bowhead whale calls based on previous studies^18^.

#### Sound propagation modeling

Acoustic modeling was used to predict the sound transmission loss of bowhead FM calls under open-water and ice-covered conditions within a 1 km resolution spatial grid of 40 km radius about each recording site. Transmission loss (TL) grids were created using the freely-available Acoustics Toolbox User Interface and Post-processor (AcTUP v 2.2L)^61^, which provides a Matlab-based graphical user interface for the Acoustics Toolbox (HLS Inc. San Diego, CA). The RAMGEO parabolic equation model^62^ was used since it is well suited for shallow water environments and for frequencies < 1 kHz^63^ and can incorporate water column, seabed, sea surface and sea ice input parameters. Sound speed profiles were selected from hydrographic data collected in ice-covered and open-water conditions (Fig. 10). The sea ice layer was assumed to be uniform first-year ice with no ridging, layer thickness 2.7 m, underside RMS roughness 1.38 m^64^, compressional speed 3000 m/s, and shear speed 1800 m/s^65^.

Transmission loss models were calculated along transects at 22.5° angular intervals originating at the recording site. Models were executed in one Hz steps from 80 to 180 Hz with source depth 26 m below the sea-air surface and receiver depth 10 m above the seafloor. The models were incoherently averaged across frequency (Alexander et al. 2013)^63^ and a 100-point moving average was applied with range, to smooth each transect. Using linear interpolation between transect points, transmission loss was estimated within a 1 km resolution grid centered on each recording site. Grid locations were mapped to geographic coordinates using a polar stereographic projection, yielding 40 km radius transmission loss grids for each site and ice condition. An additional range-dependent loss term of − 0.26 dB/km was applied to all locations in the transmission loss grids to approximate excess loss observed for the Chukchi Shelf ^31^.

#### Monte Carlo detection probability simulation

A simulation method was used to estimate call detection probability for different ocean noise levels and ice states. Seven ambient noise levels were used from 50 to 85 dB re μPa^2^ in 5 dB steps. For each noise level, two simulations were run using the modeled transmission loss grids for open-water and ice-covered states. For each combination of site, noise level, and ice state, the simulation randomly selected 100,000 bowhead call source level values and placed them within the 40 km radius grid. Source levels were randomly generated from a probability distribution fit to source level estimates for bowhead calls reported by Cummings and Holliday^46^ (157, ± 10 dB re 1 μPa @ 1 m). Each 1 km by 1 km cell in the detection grid was scored with the proportion of detected calls, those having a received level at least 2 dB above the noise level. The detection probability, *P̂*, was calculated as the proportion of detected to total simulated calls originating from that location. The mean detection probability for each site, ice condition, and noise level was calculated by weighted average of the detection probability values for all locations in the 40 km radius grid. Finally, a spline interpolation was used to fit a curve to detection probability as a function of noise level for both ice conditions.

#### Upslope and downslope sound propagation and detection probability

To examine patterns in sound transmission loss and detection probability with changing bathymetry and ice state, we identified azimuths for each site to represent upslope and downslope propagation. True bearings between 22.5 and 67.5° from both sites were defined as downslope and bearings between 202.5 and 247.5° were defined as upslope. Since the shelf site was located directly upslope from the slope site, these azimuthal definitions of up and downslope propagation were the same for both recording locations. Transmission losses and detection probabilities were averaged for all model locations within the up and downslope sectors for distances from 0 to 20 km and 20–40 km to compare relatively long and short-range estimates for the locations with and without sea ice.

### Estimating density of occurrence

Using the acoustic data recorded in the northeast Chukchi Sea (Fig. 2), the detection probability for each hour of data was estimated using the detection probability from the simulation for the measured 50th percentile noise level and the corresponding mean daily sea ice state for that hour. We calculated the hourly 10th, 50th, and 90th percentile received levels within the 80–180 Hz bowhead call frequency band, using 5 s averages from the LTSA. The first and last 15 s from each 75 s raw data file were excluded to remove instrumental self-noise associated with writing to hard disk. Assuming no false detections and a unity calling probability during each one-hour period, the daily detection density, $\hat{D}_{T}$, becomes,

5 $$\hat{D}_{T} = \frac{{\hat{N}_{T} }}{{\pi \hat{P}_{T} w^{2 } }}$$

where $\hat{N}_{T}$ is the number of hours per day with acoustic detections and $\hat{P}_{T}$ is the average detection probability of all hours in day *T*. To evaluate uncertainty in $\hat{D}$ due to noise, detection probability was also calculated for the hourly averaged 10th and 90th percentiles and input to Eq. 5 to yield lower and upper bounds, $\hat{D}_{10} {\text{and}} \hat{D}_{90}$, for detection density. A threshold of 20% ice cover was set to determine whether the detection probability function for the ice-covered or open-water model would be used for each hour.

## References

[1] Stirling I, Calvert W, Cleator H (1983). Underwater vocalizations as a tool for studying the distribution and relative abundance of wintering pinnipeds in the High Arctic. Arctic.

[2] Sirovic A, Hildebrand JA, Wiggins SM, McDonald MA, Moore SE, Thiele D (2004). Seasonality of blue and fin whale calls and the influence of sea ice in the Western Antarctic Peninsula. Deep Sea Res. II.

[3] Jones JM, Thayre BJ, Roth EH, Mahoney M, Sia I, Merculief K, Jackson C, Zeller C, Clare M, Bacon A, Weaver S, Gentes Z, Stirling I, Wiggins SM, Hildebrand JA (2014). Ringed, bearded, and ribbon seal vocalizations north of barrow, Alaska: Seasonal presence and relationship with sea ice. Arctic.

[4] Clark CW, Berchok CL, Blackwell SB, Hannay DE, Jones J, Ponirakis D, Stafford KM (2015). A year in the acoustic world of bowhead whales in the Bering, Chukchi and Beaufort seas. Prog. Oceanogr..

[5] Marques TA, Munger L, Thomas L, Wiggins S, Hildebrand JA (2011). Estimating North Pacific right whale Eubalaena japonica density using passive acoustic cue counting. Endangered Species Res..

[6] Hildebrand JA, Baumann-Pickering S, Frasier KE, Trickey JS, Merkens KP, Wiggins SM, McDonald MA, Garrison LP, Harris D, Marques TA, Thomas L (2015). Passive acoustic monitoring of beaked whale densities in the Gulf of Mexico. Sci. Rep..

[7] von Benda-Beckmann AM, Thomas L, Tyack PL, Ainslie MA (2018). Modelling the broadband propagation of marine mammal echolocation clicks for click-based population density estimates. J. Acoust. Soc. Am..

[8] Hildebrand JA, Frasier KE, Baumann-Pickering S, Wiggins SM, Merkens KP, Garrison LP, Soldevilla MS, McDonald MA (2019). Assessing seasonality and density from passive acoustic monitoring of signals presumed to be from pygmy and dwarf sperm whales in the Gulf of Mexico. Front. Mar. Sci..

[9] Marques TA, Thomas L, Martin SW, Mellinger DK, Ward JA, Moretti DJ, Harris D, Tyack PL (2013). Estimating animal population density using passive acoustics. Biol. Rev..

[10] Helble TA, D’Spain GL, Campbell GS, Hildebrand JA (2013). Calibrating passive acoustic monitoring: Correcting humpback call detections for site-specific and time-dependent environmental characterisitcs. J. Acoust. Soc. Amer..

[11] Frasier KE, Wiggins SM, Harris D, Marques TA, Thomas L, Hildebrand JA (2016). Delphinid echolocation click detection probability on near-seafloor sensors. J. Acoust. Soc. Am..

[12] Helble TA, D’Spain GL, Hildebrand JA, Campbell GS, Campbell RL, Heaney KD (2013). Site specific probability of passive acoustic detection of humpback whale calls from single fixed hydrophones. J. Acoust. Soc. Am..

[13] Larsen Tempel JT, Wise S, Osborne TQ, Sparks K, Atkinson S (2021). "Life without ice: Perceptions of environmental impacts on marine resources and subsistence users of St. Lawrence Island. Ocean Coast. Manag..

[14] Clark CW, Johnson JH (1984). The sounds of the bowhead whale, Balaena mysticetus, during the spring migrations of 1979 and 1980. Can. J. Zool..

[15] Ashjian, C. J., Braund, S. R., Campbell, R. G., George, J. C., Kruse, J., Maslowski, W., Moore, S. E., Nicolson, C. R., Okkonen, S. R., & Sherr, B. F. Climate variability, oceanography, bowhead whale distribution, and Iñupiat subsistence whaling near Barrow, Alaska, Arctic 179–194 (2010).

[16] Moore SE, Clarke JT (1993). Bowhead whale fall distribution and relative abundance in relation to oil and gas lease areas in the northeastern Chukchi Sea. Polar Rec..

[17] Reeves R, Rosa C, George JC, Sheffield G, Moore M (2012). "Implications of Arctic industrial growth and strategies to mitigate future vessel and fishing gear impacts on bowhead whales. Mar. Policy.

[18] Blackwell, S. B., Richardson, W., Greene Jr, C., & Streever, B. Bowhead whale (Balaena mysticetus) migration and calling behaviour in the Alaskan Beaufort Sea, Autumn 2001–04: An acoustic localization study, Arctic 255–270 (2007).

[19] Mathias, D., Thode, A., Blackwell, S. B., & Greene, C. Computer-aided classification of bowhead whale call categories for mitigation monitoring. In *New Trends for Environmental Monitoring Using Passive Systems*, Hyeres, French Riviera 1–6 (2008).

[20] Delarue J, Laurinolli M, Martin B (2009). Bowhead whale (Balaena mysticetus) songs in the Chukchi Sea between October 2007 and May 2008. J. Acoust. Soc. Am..

[21] Moore SE, Stafford KM, Munger LM (2010). Acoustic and visual surveys for bowhead whales in the western Beaufort and far northeastern Chukchi seas. Deep Sea Res. Part II.

[22] Ballard MS, Badiey M, Sagers JD, Colosi JA, Turgut A, Pecknold S, Lin Y-T, Proshutinsky A, Krishfield R, Worcester PF, Dzieciuch MA (2020). Temporal and spatial dependence of a yearlong record of sound propagation from the Canada Basin to the Chukchi Shelf. J. Acoust. Soc. Am..

[23] Duda TF, Zhang WG, Lin Y-T (2021). Effects of Pacific Summer Water layer variations and ice cover on Beaufort Sea underwater sound ducting. J. Acoust. Soc. Am..

[24] Diachok OI, Winokur RS (1974). Spatial variability of underwater ambient noise at the Arctic ice-water boundary. J. Acoust. Soc. Am..

[25] Diachok OI (1976). Effects of sea-ice ridges on sound propagation in the Arctic Ocean. J. Acoust. Soc. Am..

[26] Yang T, Votaw CW (1981). Under ice reflectivities at frequencies below 1 kHz. J. Acoust. Soc. Am..

[27] Milne AR, Ganton JH (1964). Ambient Noise under Arctic-Sea Ice. J. Acoust. Soc. Am..

[28] Brown JR, Milne AR (1967). Reverberation under Arctic Sea-Ice. J. Acoust. Soc. Am..

[29] Duckworth G, LePage K, Farrell T (2001). Low-frequency long-range propagation and reverberation in the central Arctic: Analysis of experimental results. J. Acoust. Soc. Am..

[30] Jensen FB, Kuperman WA (1983). Optimum frequency of propagation in shallow water environments. J. Acoust. Soc. Am..

[31] Keen KA, Thayre BJ, Hildebrand JA, Wiggins SM (2018). Seismic airgun sound propagation in Arctic Ocean waveguides. Deep Sea Res. I.

[32] Greene CR, Buck BM (1964). Arctic ocean ambient noise. J. Acoust. Soc. Am..

[33] Roth EH, Hildebrand JA, Wiggins SM, Ross D (2012). Underwater ambient noise on the Chukchi Sea continental slope from 2006–2009. J. Acoust. Soc. Am..

[34] Kinda GB, Simard Y, Gervaise C, Mars JI, Fortier L (2015). Arctic underwater noise transients from sea ice deformation: Characteristics, annual time series, and forcing in Beaufort Sea. J. Acoust. Soc. Am..

[35] Hildebrand JA, Frasier KE, Baumann-Pickering S, Wiggins SM (2021). An empirical model for wind-generated ocean noise. J. Acoust. Soc. Am..

[36] Farmer DM, Xie Y (1988). The sound of cracking sea ice. J. Acoust. Soc. Am..

[37] Bassett C, Thomson J, Dahl PH, Polagye B (2014). Flow-noise and turbulence in two tidal channels. J. Acoust. Soc. Am..

[38] Chapman RP, Harris J (1962). Surface backscattering strengths measured with explosive sound sources. J. Acoust. Soc. Am..

[39] Gauss, R. C., Fialkowski, J. M., & Wurmser, D. A low- and mid-frequency bistatic scattering model for the ocean surface. In Proceedings of OCEANS 2005 MTS/IEEE, Vol. 2 1738–1744 (2005)

[40] Jin G, Lynch JF, Pawlowicz R, Worcester P (1994). Acoustic scattering losses in the Greenland Sea marginal ice zone during the 1988–89 tomography experiment. J. Acoust. Soc. Am..

[41] Citta JJ, Quakenbush LT, Okkonen SR, Druckenmiller ML, Maslowski W, Clement-Kinney J, George JC, Brower H, Small RJ, Ashjian CJ, Harwood LA, Heide-Jorgensen MP (2015). Ecological characteristics of core-use areas used by Bering-Chukchi-Beaufort (BCB) bowhead whales, 2006–2012. Prog. Oceanogr..

[42] Thode AM, Blackwell SB, Conrad AS, Kim KH, Macrander AM (2017). Decadal-scale frequency shift of migrating bowhead whale calls in the shallow Beaufort Sea. J. Acoust. Soc. Am..

[43] Wiggins, S. M., & Hildebrand, J. A. High-frequency acoustic recording package (HARP) for broad-band, long-term marine mammal monitoring. In International Symposium on Underwater Technology 2007 and International Workshop on Scientific use of Submarine Cables & Related Technologies 2007. Institute of Electrical and Electronics Engineers, Tokyo, Japan 551–557 (2007).

[44] Marques TA, Thomas L, Ward J, DiMarzio N, Tyack PL (2009). Estimating cetacean population density using fixed passive acoustic sensors: An example with Blainville’s beaked whales. J. Acoust. Soc. Am..

[45] Küsel ET, Mellinger DK, Thomas L, Marques TA, Moretti D, Ward J (2011). Cetacean population density estimation from single fixed sensors using passive acoustics. J. Acoust. Soc. Am..

[46] Cummings WC, Holliday DV (1987). Sounds and source levels from bowhead whales off Pt. Barrow, Alaska. J. Acoust. Soc. Am..

[47] Thode AM, Blackwell SB, Seger KD, Conrad AS, Kim KH, Michael Macrander A (2016). Source level and calling depth distributions of migrating bowhead whale calls in the shallow Beaufort Sea. J. Acoust. Soc. Am..

[48] Blackwell SB, McDonald TL, Kim KH, Aerts LAM, Richardson WJ, Greene JCR, Streever B (2012). Directionality of bowhead whale calls measured with multiple sensors. Mar. Mammal Sci..

[49] Markus T, Stroeve JC, Miller J (2009). Recent changes in Arctic sea ice melt onset, freezeup, and melt season length. J. Geophys. Res. Oceans.

[50] Kwok R, Cunningham G (2015). Variability of Arctic sea ice thickness and volume from CryoSat-2. Philos. Trans. R. Soc. A Math. Phys. Eng. Sci..

[51] Krishfield R, Toole J, Proshutinsky A, Timmermans M-L (2008). Automated ice-tethered profilers for seawater observations under pack ice in all seasons. J. Atmos. Oceanic Tech..

[52] Gong D, Pickart RS (2015). Summertime circulation in the eastern Chukchi Sea. Deep Sea Res. Part II.

[53] Meng X, Li G, Han G, Kan G (2015). Sound velocity and related properties of seafloor sediments in the Bering Sea and Chukchi Sea. Acta Oceanol. Sin..

[54] Toole JM, Krishfield RA, Timmermans M-L, Proshutinsky A (2011). The ice-tethered profiler: Argo of the Arctic. Oceanography.

[55] Millero FJ, Feistel R, Wright DG, McDougall TJ (2008). The composition of Standard Seawater and the definition of the Reference-Composition Salinity Scale. Deep Sea Res. Part I.

[56] Kutschale H (1961). Long-range sound transmission in the Arctic Ocean. J. Geophys. Res..

[57] Jakobsson M, Mayer L, Coakley B, Dowdeswell JA, Forbes S, Fridman B, Hodnesdal H, Noormets R, Pedersen R, Rebesco M (2012). The international bathymetric chart of the Arctic Ocean (IBCAO) version 3.0. Geophys. Res. Lett..

[58] Warner GA, Dosso SE, Dettmer J, Hannay DE (2015). Bayesian environmental inversion of airgun modal dispersion using a single hydrophone in the Chukchi Sea. J. Acoust. Soc. Am..

[59] Pang X, Pu J, Zhao X, Ji Q, Qu M, Cheng Z (2018). Comparison between AMSR2 sea ice concentration products and pseudo-ship observations of the Arctic and Antarctic sea ice edge on cloud-free days. Remote Sens..

[60] Kahru, M. Windows image manager: Image display and analysis program for Microsoft Windows with special features for satellite images (2001).

[61] Duncan AJ, Maggi AL (2006). A consistent, user friendly interface for running a variety of underwater acoustic propagation codes. Proc. Acoust..

[62] Collins MD (1993). A split-step Padé solution for the parabolic equation method. J. Acoust. Soc. Am..

[63] Alexander, P., Duncan, A., Bose, N., & Smith, D. Modelling acoustic transmission loss due to sea ice cover. *Acoust. Aust.* **41** (2013).

[64] Goff JA (1995). Quantitative analysis of sea ice draft: 1. Methods for stochastic modeling. J. Geophys. Res. Oceans.

[65] Gavrilov AN, Mikhalevsky PN (2006). Low-frequency acoustic propagation loss in the Arctic Ocean: Results of the Arctic climate observations using underwater sound experiment. J. Acoust. Soc. Am..

